# An IMM Extension of RBPMF for Terrain-Referenced Navigation Under Radar Return Ambiguity

**DOI:** 10.3390/s26196024

**Published:** 2026-09-23

**Authors:** Kyung Jun Han, Chang-Ky Sung, ByungSu Park, Taeyun Kim, Chan Gook Park

**Affiliations:** 1 Department of Mechanical and Aerospace Engineering, Seoul National University, 1 Gwanak-ro, Gwanak-gu, Seoul 08826, Republic of Korea; 2 4th R&D Institute, Agency for Defense Development, Yuseong, Daejeon 34186, Republic of Korea

**Keywords:** terrain-referenced navigation, Rao–Blackwellized point-mass filter, interacting multiple model, radar-altimeter uncertainty, mode-dependent measurement noise, Bayesian filtering

## Abstract

**Highlights:**

**What are the main findings?**

- Grid-resolved posterior mixing enables deterministic interaction of complete mode-conditioned Rao–Blackwellized point-mass filter posteriors.
- Monte Carlo simulations show lower horizontal-position and altitude RMSEs than the evaluated single-model and fixed-mixture RBPMF baselines.

**What are the implications of the main findings?**

- The findings highlight the value of jointly addressing radar-return ambiguity and multimodal position uncertainty in navigation-filter design.
- The results support more robust terrain-referenced navigation under ground–canopy return ambiguity, particularly over weakly informative terrain.

**Abstract:**

Terrain-referenced navigation (TRN) relies on nonlinear terrain matching, which can produce multimodal position uncertainty that should be retained during estimation. Accuracy can deteriorate when the relative probabilities of ground and canopy returns vary over time because a fixed measurement model cannot adapt to the changing return regime. To address this problem, an interacting multiple-model (IMM) extension of the Rao–Blackwellized point-mass filter (RBPMF) is developed, yielding an IMM-RBPMF that adapts between measurement hypotheses while retaining the non-Gaussian navigation posterior. The extension uses grid-resolved posterior mixing to remap complete mode-conditioned RBPMF representations onto a common grid; nonlinear-state point masses are combined using global IMM interaction probabilities, whereas conditional Gaussian components are combined using state-dependent local responsibilities. Monte Carlo simulations over rough and rough–flat–rough terrain compare the proposed filter with single-model and fixed-mixture RBPMFs. Relative to the best baseline for each metric, the filter reduces overall horizontal-position root-mean-square error (RMSE) by up to 44% and altitude RMSE by 53%, demonstrating improved robustness to ambiguous radar returns and weakly informative terrain.

## 1. Introduction

Accurate and reliable navigation is essential for autonomous and safety-critical vehicles operating in environments where global navigation satellite system (GNSS) signals are unavailable, degraded, or intentionally interfered with. Although an inertial navigation system (INS) provides self-contained navigation information, its position, velocity, and attitude errors inevitably accumulate over time. Terrain-referenced navigation (TRN) constrains this error growth by comparing terrain-related measurements obtained from onboard altimetry with a pre-stored digital elevation model (DEM). Because the DEM-based measurement is highly nonlinear with respect to horizontal position and may admit multiple plausible position hypotheses over repetitive or weakly varying terrain, TRN is naturally formulated as a nonlinear and potentially non-Gaussian Bayesian estimation problem [1,2].

Particle filters (PFs) and point-mass filters (PMFs) are representative global Bayesian filtering approaches to TRN. PFs approximate nonlinear and non-Gaussian posterior densities using weighted random samples, or particles; however, with a finite number of particles, they may suffer from particle degeneracy, sample impoverishment, and run-to-run variability [2,3]. In contrast, PMFs approximate the posterior density on a deterministic grid, providing repeatable estimation results and reliable retention of low-probability state hypotheses [2]. PMFs nevertheless face finite-grid approximation error because estimation quality depends on how accurately the grid represents and propagates the conditional density. Like PFs, PMFs are also subject to the curse of dimensionality; for conventional full-grid PMFs, this limitation is particularly restrictive because the number of grid points grows exponentially with the state dimension and the grid-based Chapman–Kolmogorov time propagation becomes increasingly computationally demanding. Adaptive grid and support designs, reliable time-propagation schemes, and Gaussian-mixture-assisted grid construction improve finite-grid accuracy, whereas efficient convolution methods reduce the cost of large-grid operations [4,5,6,7,8,9]. These approaches alleviate, but do not eliminate, dimensional scaling. Rao–Blackwellized PMFs and related marginalized or hybrid formulations address this limitation more directly by applying the global nonlinear estimator only to a subset of the state variables [10,11,12]. Collectively, these developments have established the PMF as an important deterministic alternative to the PF for TRN.

Beyond nonlinear state estimation, practical radar-altimeter-based TRN must also account for radar return ambiguity. A DEM generally represents bare-ground elevation, whereas an actual radar return may originate from the ground, vegetation canopy, or another elevated object within the radar footprint. These alternative return sources can induce distinct measurement-error distributions with different means and variances. As the dominant return source changes over time, the radar-altimeter error distribution can become multimodal and time varying [13,14]. A fixed Gaussian-mixture likelihood can represent multiple error components within an individual measurement update. However, when a single state posterior with fixed mixture weights is maintained, the temporal association between a persistent radar-return mode and its corresponding navigation-state posterior is not explicitly preserved [14]. Ground- and canopy-return conditions that persist across consecutive measurements are therefore more naturally represented as switching measurement models.

The interacting multiple-model (IMM) algorithm is a widely used recursive framework for estimation under Markovian model switching [15]. It predicts mode probabilities, interacts mode-conditioned state estimates, and updates parallel filters using the corresponding mode-dependent models. For nonlinear and non-Gaussian systems, various interacting multiple-model particle filter (IMM-PF) formulations have been developed for state estimation [16,17,18,19,20], including applications to positioning [18] and target tracking [19]. In TRN, our previous study [14] represented ground- and canopy-return conditions as discrete measurement modes and developed an IMM-PF with a classification-error-minimizing mode update. Although the resulting IMM-PF improved estimation accuracy and robustness relative to the conventional IMM-PF, its stochastic particle representation remained subject to finite-sample and resampling-related effects, which can hinder the reliable retention of low-probability alternative position hypotheses [2,3].

By contrast, IMM extensions of deterministic PMFs have rarely been reported. Copp and Subbarao [21] employed a bank of two-dimensional PMFs for altitude-bias estimation in TRN within a multiple-model adaptive estimation (MMAE) framework, without IMM interaction. Likewise, point-mass and deterministic-grid multiple-model estimators developed for model identification [22] and target tracking [23] were not formulated within the IMM framework. Thus, to the best of the authors’ knowledge, an interacting multiple-model point-mass filter (IMM-PMF) framework that recursively interacts mode-conditioned PMF posteriors has not previously been developed, particularly for TRN.

For TRN, however, filling this gap with a full-dimensional IMM-PMF is computationally demanding because the radar-altimeter measurement couples horizontal position and altitude. Augmenting a conventional two-dimensional horizontal-position PMF with altitude and other INS error states rapidly increases the number of grid points. The Rao–Blackwellized point-mass filter (RBPMF) mitigates this dimensionality problem by retaining a deterministic grid only for the nonlinear horizontal-position states, while representing altitude and the remaining navigation-error states by grid-conditioned Gaussian distributions [10]. Thus, a practical IMM extension for the augmented INS/TRN navigation-error state is naturally formulated as an interacting multiple-model Rao–Blackwellized point-mass filter (IMM-RBPMF).

Accordingly, this paper extends the interacting multiple-model framework to Rao–Blackwellized point-mass filtering for TRN under radar return ambiguity, yielding the proposed IMM-RBPMF. At each epoch, the proposed algorithm (i) updates mode-conditioned RBPMFs for the ground- and canopy-return hypotheses, together with their corresponding mode probabilities, (ii) interacts the complete mode-conditioned RBPMF representations across modes, and (iii) propagates the resulting mixed RBPMFs to the next epoch. The standard marginalized likelihood is used for the navigation-state update, whereas the truncated likelihood from [14] is retained for the mode-probability update.

The main contributions of this study are threefold. First, an IMM extension of Rao–Blackwellized point-mass filtering is formulated for nonlinear multiple-model estimation within a deterministic framework. Second, a deterministic IMM-interaction procedure, termed grid-resolved posterior mixing, is developed to remap and combine complete mode-conditioned RBPMF representations on a common nonlinear-state grid. Third, the proposed algorithm is applied to TRN with ground- and canopy-return uncertainty and validated through comparative Monte Carlo simulations in terms of accuracy, robustness, and computational cost.

The remainder of this paper is organized as follows. Section 2 presents the Rao–Blackwellized TRN model with mode-dependent radar-altimeter uncertainty. Section 3 derives the proposed IMM-RBPMF recursion and describes its deterministic grid-based implementation. Section 4 presents the simulation setup. Section 5 presents the results and discussion. Section 6 concludes the paper.

## 2. Problem Formulation

### 2.1. Rao–Blackwellized TRN Model with Mode-Dependent Radar-Altimeter Uncertainty

Following the mixed linear/nonlinear formulation in [10,11], the navigation-error state is partitioned as

(1) $${x_{k}=\left[(x_{k}^{n}{)}^{T},(x_{k}^{l}{)}^{T}\right]}^{T},$$

(2) $${x_{k}^{n}=\left[\delta p_{E,k},\delta p_{N,k}\right]}^{T},$$

(3) $${x_{k}^{l}=\left[\delta h_{k},\delta v_{E,k},\delta v_{N,k},\delta v_{U,k},\phi_{E,k},\phi_{N,k},\phi_{U,k}\right]}^{T}.$$

Here, $x_{k}^{n}$ contains the horizontal-position errors and $x_{k}^{l}$ contains the altitude, velocity, and attitude errors. The former are treated as nonlinear because they enter the DEM measurement through the terrain-height function. Conditioned on $x_{k}^{n}$, the remaining states are represented by Gaussian statistics, while their coupling is retained through the dynamics, process noise, and measurement update.

The radar-return condition is represented by a first-order Markov mode $\theta_{k}\in \Theta =\{\theta^{1},\ldots ,\theta^{M}\}$. The transition probability is

(4) $$\pi_{ij}=P(\theta_{k+1}=\theta^{j}|\theta_{k}=\theta^{i}).$$

The transition probabilities satisfy $\sum_{j=1}^{M}\pi_{ij}=1$. The mode $\theta_{k}$ governs the measurement at time k. In this study, we set M=2, corresponding to ground and canopy returns. The transition matrix $\Pi =\left[\pi_{ij}\right]$ is used as a filter-side persistence prior that regularizes the recursive mode probabilities; it is not interpreted as the physical surface-cover transition law. Conditioned on $\theta_{k}$, the mode transition is assumed independent of the continuous state and measurement history.

The INS error dynamics are expressed in the mixed linear/nonlinear form as

(5) $$\left[\begin{array}{c} x_{k+1}^{n}\\ x_{k+1}^{l} \end{array}\right]=\left[\begin{array}{c} f_{k}^{n}\left(x_{k}^{n}\right)\\ f_{k}^{l}\left(x_{k}^{n}\right) \end{array}\right]+\left[\begin{array}{c} F_{k}^{n}\left(x_{k}^{n}\right)\\ F_{k}^{l}\left(x_{k}^{n}\right) \end{array}\right]x_{k}^{l}+w_{k},$$

(6) $$w_{k}=\left[\begin{array}{c} w_{k}^{n}\\ w_{k}^{l} \end{array}\right]∼N\left(0,Q_{k}\right).$$

Here, $f_{k}^{n}\left(\cdot \right)\in R^{2}$ and $f_{k}^{l}\left(\cdot \right)\in R^{7}$ denote the terms independent of $x_{k}^{l}$, while $F_{k}^{n}\left(\cdot \right)\in R^{2\times 7}$ and $F_{k}^{l}\left(\cdot \right)\in R^{7\times 7}$ are the coefficient matrices multiplying $x_{k}^{l}$. For the linear time-varying INS error model, (5) can be written as

(7) $$x_{k+1}=\underset{A_{k}\in R^{9\times 9}}{\underbrace{\left[\begin{array}{cc} A_{k}^{nn} & A_{k}^{nl}\\ A_{k}^{ln} & A_{k}^{ll} \end{array}\right]}}x_{k}+w_{k},$$

where $f_{k}^{n}\left(x_{k}^{n}\right)=A_{k}^{nn}x_{k}^{n}$, $f_{k}^{l}\left(x_{k}^{n}\right)=A_{k}^{ln}x_{k}^{n}$, $F_{k}^{n}\left(x_{k}^{n}\right)=A_{k}^{nl}$, and $F_{k}^{l}\left(x_{k}^{n}\right)=A_{k}^{ll}$. The blocks of $A_{k}$ are evaluated from the nominal navigation solution, and their explicit expressions for the nine-state east-north-up (ENU) model are provided in Appendix A. The dynamics are common to all modes because the discrete mode represents radar-altimeter uncertainty rather than a change in vehicle motion.

Let $Y_{0:k}=\left\{y_{0},\ldots ,y_{k}\right\}$. For $\theta_{k}=\theta^{i}$, the terrain-height measurement is modeled as

(8) $$y_{k}=h_{k}(x_{k}^{n})+H_{k}(x_{k}^{n})x_{k}^{l}+v_{k}^{i},$$

(9) $$v_{k}^{i}∼N(m_{v,k}^{i},R_{k}^{i}).$$

Here, $h_{k}\left(\cdot \right)$ is the nonlinear DEM-dependent term, $H_{k}\left(\cdot \right)$ is the sensitivity to $x_{k}^{l}$, and $m_{v,k}^{i}$ and $R_{k}^{i}$ are the mode-dependent radar-error mean and variance. Thus, mode dependence enters the present model only through the measurement-error distribution.

### 2.2. Posterior Factorization and RBPMF Representation

The joint filtering density is factorized as

(10) $$p(x_{k}^{n},x_{k}^{l},\theta_{k}|Y_{0:k})=p(x_{k}^{l}|x_{k}^{n},\theta_{k},Y_{0:k})p(x_{k}^{n}|\theta_{k},Y_{0:k})p(\theta_{k}|Y_{0:k}).$$

For mode $\theta^{i}$, the nonlinear-state posterior is approximated on a deterministic PMF grid:

(11) $$p(x_{k}^{n}|\theta_{k}=\theta^{i},Y_{0:k})\approx \sum_{g=1}^{G}w_{k|k}^{i,[g]}\delta \left(x_{k}^{n}-\xi_{k}^{i,[g]}\right).$$

Here, $\delta \left(\cdot \right)$ denotes the two-dimensional Dirac delta distribution and the probability masses satisfy $w_{k|k}^{i,[g]}\geq 0$ and $\sum_{g=1}^{G}w_{k|k}^{i,[g]}=1$. The grid point $\xi_{k}^{i,\left[g\right]}$ represents the g-th candidate horizontal-position-error hypothesis in the east–north plane under mode $\theta^{i}$, and $w_{k|k}^{i,\left[g\right]}$ is the posterior probability mass assigned to that hypothesis. At each grid point, the conditional linear-state posterior is represented by

(12) $$p(x_{k}^{l}|x_{k}^{n}=\xi_{k}^{i,[g]},\theta_{k}=\theta^{i},Y_{0:k})\approx N\left(x_{k}^{l};{\hat{x}}_{k|k}^{i,l,[g]},P_{k|k}^{i,l,[g]}\right).$$

The filtered (a posteriori) mode probability is

(13) $$\mu_{k}^{i}=P(\theta_{k}=\theta^{i}|Y_{0:k}),$$

where $\sum_{i=1}^{M}\mu_{k}^{i}=1$. Throughout this paper, $\mu_{k}^{i}$ denotes the filtered mode probability after incorporating $y_{k}$, whereas $\mu_{k|k-1}^{i}=P\left(\theta_{k}=\theta^{i}|Y_{0:k-1}\right)$ denotes the predicted (a priori) mode probability. For compact notation, the mode-conditioned RBPMF representation is defined as

(14) $$D_{k|k}^{i}={\left\{\xi_{k}^{i,[g]},w_{k|k}^{i,[g]},{\hat{x}}_{k|k}^{i,l,[g]},P_{k|k}^{i,l,[g]}\right\}}_{g=1}^{G}.$$

The complete posterior is represented by ${\left\{\left(\mu_{k}^{i},D_{k|k}^{i}\right)\right\}}_{i=1}^{M}$. Although all modes use G grid points, their supports and locations may differ. Section 3 develops the IMM interaction required to remap and mix these mode-conditioned RBPMF representations.

## 3. Proposed IMM-RBPMF

At each measurement epoch, the proposed filter updates the mode-conditioned RBPMFs and mode probabilities, interacts the filtered representations for each destination mode, and propagates them to the next epoch, as summarized in Figure 1. The measurement and propagation steps follow the conventional RBPMF [10]. The proposed recursion uses the truncated-likelihood mode update developed in [14], while its principal new element is the deterministic interaction of complete mode-conditioned RBPMF representations defined on potentially different nonlinear-state grids.

### 3.1. Measurement and Mode Update

Let $D_{k|k-1}^{i}$ and $\mu_{k|k-1}^{i}$ denote the predicted RBPMF representation and mode probability for $\theta^{i}$, respectively. At grid point $\xi_{k}^{i,\left[g\right]}$, define

$$h_{k}^{i,[g]}\equiv h_{k}(\xi_{k}^{i,[g]}),H_{k}^{i,[g]}\equiv H_{k}(\xi_{k}^{i,[g]}).$$

After marginalizing the conditionally Gaussian state, the likelihood is

(15) $$l_{k}^{i,[g]}=N\left(y_{k};\;h_{k}^{i,[g]}+H_{k}^{i,[g]}{\hat{x}}_{k|k-1}^{i,l,[g]}+m_{v,k}^{i},\;S_{k}^{i,[g]}\right),$$

where the innovation variance is

(16) $$S_{k}^{i,[g]}=H_{k}^{i,[g]}P_{k|k-1}^{i,l,[g]}{\left(H_{k}^{i,[g]}\right)}^{T}+R_{k}^{i}.$$

The nonlinear-state masses are updated as

(17) $$w_{k|k}^{i,[g]}=\frac{w_{k|k-1}^{i,[g]}l_{k}^{i,[g]}}{\sum_{s=1}^{G}w_{k|k-1}^{i,[s]}l_{k}^{i,[s]}}.$$

The conditional mean and covariance at each grid point are updated using the standard Kalman measurement update associated with the measurement model in Equations (8) and (9).

For mode estimation, define the uncentered measurement residual and the corresponding truncated-likelihood mode update as

(18) $$\nu_{k}^{i,[g]}=y_{k}-h_{k}^{i,[g]}-H_{k}^{i,[g]}{\hat{x}}_{k|k-1}^{i,l,[g]},$$

(19) $$\Lambda_{k,M}^{i}=\sum_{g=1}^{G}w_{k|k-1}^{i,[g]}p_{TL}^{i,[g]}\left(\nu_{k}^{i,[g]};m_{v,k}^{i},S_{k}^{i,[g]}\right),$$

(20) $$\mu_{k}^{i}=\frac{\Lambda_{k,M}^{i}\mu_{k|k-1}^{i}}{\sum_{r=1}^{M}\Lambda_{k,M}^{r}\mu_{k|k-1}^{r}}.$$

Under mode $\theta^{i}$, the uncentered residual in Equation (18) has mean $m_{v,k}^{i}$ and variance $S_{k}^{i,\left[g\right]}$. Accordingly, both quantities are incorporated into the normalized truncated likelihood in Equation (19). Here, $p_{TL}^{i,[g]}\left(\nu_{k}^{i,[g]};m_{v,k}^{i},S_{k}^{i,[g]}\right)$ denotes the normalized truncated Gaussian likelihood, whose truncation boundary and normalization follow the classification-error-minimization procedure in [14]. Equation (19) gives the mode evidence, and Equation (20) yields the filtered mode probability. Thus, the ordinary marginalized likelihood in Equation (15) updates the mode-conditioned navigation-state distribution, whereas the truncated evidence in Equation (19) updates the mode probabilities through Equation (20). Because the state and mode updates use different likelihood constructions, the resulting mode-weighted RBPMF representation is an approximate filtering distribution rather than the exact posterior under a single common likelihood, consistent with the modified mode-update framework of [14].

The combined filtered-state estimate is

(21) $${\hat{x}}_{k|k}=\sum_{i=1}^{M}\mu_{k}^{i}\sum_{g=1}^{G}w_{k|k}^{i,\left[g\right]}\left[\begin{array}{c} \xi_{k}^{i,\left[g\right]}\\ {\hat{x}}_{k|k}^{i,l,\left[g\right]} \end{array}\right],$$

with its covariance obtained from the corresponding conditional and between-component covariances.

### 3.2. IMM Interaction via Grid-Resolved Posterior Mixing

After the measurement update, the predicted destination-mode probability and the global interaction probability are, respectively,

(22) $$\mu_{k+1|k}^{j}=\sum_{i=1}^{M}\pi_{ij}\mu_{k}^{i},$$

(23) $$\mu_{k}^{i|j}=\frac{\pi_{ij}\mu_{k}^{i}}{\mu_{k+1|k}^{j}}.$$

For destination mode $\theta^{j}$, let the subscript 0 denote the interacted representation at time k, which serves as the mixed initial condition for propagation to k+1. The interacted joint density is

(24) $$p_{0,k}^{j}(x_{k}^{n},x_{k}^{l})\equiv p(x_{k}^{n},x_{k}^{l}|\theta_{k+1}=\theta^{j},Y_{0:k})\;=\sum_{i=1}^{M}\mu_{k}^{i|j}p(x_{k}^{n},x_{k}^{l}|\theta_{k}=\theta^{i},Y_{0:k}).$$

Its nonlinear-state marginal is

(25) $$p_{0,k}^{j}(x_{k}^{n})=\sum_{i=1}^{M}\mu_{k}^{i|j}p(x_{k}^{n}|\theta_{k}=\theta^{i},Y_{0:k}),$$

and the conditional linear-state density is

(26) $$p_{0,k}^{j}(x_{k}^{l}|x_{k}^{n})=\sum_{i=1}^{M}\gamma_{k}^{i|j}(x_{k}^{n})p(x_{k}^{l}|x_{k}^{n},\theta_{k}=\theta^{i},Y_{0:k}),$$

(27) $$\gamma_{k}^{i|j}(x_{k}^{n})=\frac{\mu_{k}^{i|j}p(x_{k}^{n}|\theta_{k}=\theta^{i},Y_{0:k})}{\sum_{r=1}^{M}\mu_{k}^{r|j}p(x_{k}^{n}|\theta_{k}=\theta^{r},Y_{0:k})}.$$

Unlike the global interaction probability $\mu_{k}^{i|j}$, the local responsibility $\gamma_{k}^{i|j}\left(x_{k}^{n}\right)$ is the source-mode probability additionally conditioned on the nonlinear state. It therefore weights the conditional Gaussian mixture using both the global IMM probability and the local nonlinear-state density. When the mode-conditioned nonlinear-state marginals coincide, $\gamma_{k}^{i|j}\left(x_{k}^{n}\right)$ reduces to $\mu_{k}^{i|j}$. Its derivation is provided in Appendix B.

Figure 2 provides an overview of grid-resolved posterior mixing, the proposed deterministic implementation of the joint-density interaction in Equations (24)–(27), with Figure 2a illustrating nonlinear-state remapping and mass mixing and Figure 2b illustrating grid-conditioned Gaussian transfer and moment matching.

Because the source-mode grids

$$\Xi_{k}^{i}={\left\{\xi_{k}^{i,\left[g\right]}\right\}}_{g=1}^{G}$$

may differ in location, support, and resolution, their PMF representations cannot be mixed point by point. Following the conventional PMF support-adaptation approach [1], each source-mode grid is first resized to its non-negligible support. Specifically, grid points satisfying $w_{k|k}^{i,\left[g\right]}>\varepsilon_{w}/G$ are retained, where 1/G is the average mass of a uniform G-point PMF and $\varepsilon_{w}=10^{-4}$ is used as the nominal setting. For a normalized source PMF, the total mass at points satisfying $w_{k|k}^{i,\left[g\right]}\leq \varepsilon_{w}/G$ is at most $\varepsilon_{w}$, providing a per-interaction upper bound on support-truncation loss. A rectangular target grid

$$\Xi_{0,k}={\left\{\xi_{0,k}^{\left[g\right]}\right\}}_{g=1}^{G}$$

is then constructed to cover the resized supports of all source modes. More sophisticated grid-design and support-management methods are available [5,6,7], but target-grid optimization is outside the scope of this study.

Let $S_{i,g}$ denote the neighboring points of source grid i used for target point $\xi_{0,k}^{\left[g\right]}$, and let $\lambda_{s,g}^{i}$ denote the corresponding bilinear-interpolation coefficients, satisfying

$$\lambda_{s,g}^{i}\geq 0,\sum_{s\in S_{i,g}}\lambda_{s,g}^{i}=1.$$

These interpolation coefficients are determined by the relative position of the target point within the surrounding source-grid cell. To account for differences in source- and target-grid cell areas, each source probability mass is first converted to a density-equivalent value, interpolated onto the target grid, and then renormalized as a target-grid probability mass:

(28) $$\rho_{k|k}^{i,\left[s\right]}=\frac{w_{k|k}^{i,\left[s\right]}}{A_{k}^{i}},$$

(29) $${\tilde{\rho }}_{k|k}^{i,\left[g\right]}=\sum_{s\in S_{i,g}}\lambda_{s,g}^{i}\rho_{k|k}^{i,\left[s\right]},$$

(30) $${\tilde{w}}_{k|k}^{i,\left[g\right]}=\frac{A_{0,k}{\tilde{\rho }}_{k|k}^{i,\left[g\right]}}{\sum_{h=1}^{G}A_{0,k}{\tilde{\rho }}_{k|k}^{i,\left[h\right]}},$$

(31) $$w_{0,k}^{j,\left[g\right]}=\sum_{i=1}^{M}\mu_{k}^{i|j}{\tilde{w}}_{k|k}^{i,\left[g\right]}.$$

Here, $A_{k}^{i}$ and $A_{0,k}$ denote the uniform cell areas of source grid i and the target grid, respectively. For a target-grid point outside the retained support of source mode i, ${\tilde{\rho }}_{k|k}^{i,\left[g\right]}$ and ${\tilde{w}}_{k|k}^{i,\left[g\right]}$ are set to zero, thereby avoiding extrapolation beyond the source grid. The normalization in Equation (30) restores unit total mass after support truncation and source-to-target remapping, after which Equation (31) mixes the remapped masses using the global IMM interaction probabilities.

The conditional means are transferred using the same interpolation coefficients, while the covariances are interpolated in the log-Euclidean domain [24]:

(32) $${\tilde{x}}_{k|k}^{i,l,[g]}=\sum_{s\in S_{i,g}}\lambda_{s,g}^{i}{\hat{x}}_{k|k}^{i,l,[s]},$$

(33) $${\tilde{P}}_{k|k}^{i,l,[g]}=exp\left[\sum_{s\in S_{i,g}}\lambda_{s,g}^{i}\log\left(P_{k|k}^{i,l,[s]}\right)\right].$$

Equations (32) and (33) interpolate the grid-conditioned Gaussian parameter fields within each source mode, whereas probabilistic mixing across source modes is performed in Equations (34)–(36). The log-Euclidean covariance interpolation preserves the symmetric positive-definite property and avoids the artificial covariance-volume swelling associated with direct Euclidean interpolation [24]. At each target point, the Gaussian-interaction stage illustrated in Figure 2b is performed using the discrete local responsibilities and moment-matched Gaussian parameters:

(34) $$\gamma_{k}^{i|j,[g]}=\frac{\mu_{k}^{i|j}{\tilde{w}}_{k|k}^{i,[g]}}{\sum_{r=1}^{M}\mu_{k}^{r|j}{\tilde{w}}_{k|k}^{r,[g]}},$$

(35) $${\hat{x}}_{0,k}^{j,l,[g]}=\sum_{i=1}^{M}\gamma_{k}^{i|j,[g]}{\tilde{x}}_{k|k}^{i,l,[g]},$$

(36) $$P_{0,k}^{j,l,[g]}=\sum_{i=1}^{M}\gamma_{k}^{i|j,[g]}\left[{\tilde{P}}_{k|k}^{i,l,[g]}+\Delta_{k}^{ij,[g]}{\left(\Delta_{k}^{ij,[g]}\right)}^{T}\right],$$

(37) $$\Delta_{k}^{ij,[g]}={\tilde{x}}_{k|k}^{i,l,[g]}-{\hat{x}}_{0,k}^{j,l,[g]}.$$

The local responsibilities in Equation (34) are recomputed at each interaction step from quantities available within the filter, rather than specified as tuning parameters. Specifically, the interaction probability $\mu_{k}^{i|j}$ is computed from the filtered mode probabilities in Equation (20) and the prescribed transition matrix using Equations (22) and (23). The remapped mass ${\tilde{w}}_{k|k}^{i,\left[g\right]}$ is obtained from the measurement-updated source masses using Equations (28)–(30). At each target-grid point, the products $\mu_{k}^{i|j}{\tilde{w}}_{k|k}^{i,\left[g\right]}$ are normalized over the source modes. Because the denominator of the local responsibility equals the destination-mode mass $w_{0,k}^{j,\left[g\right]}$, Equation (34) is evaluated only at target points where $w_{0,k}^{j,\left[g\right]}$ is nonzero. Source modes with zero remapped mass have zero local responsibility and therefore make no contribution to the mixed mean or covariance. At each target-grid point and for each destination mode, Equations (35) and (36) approximate the conditional mixture formed from the remapped components by a single Gaussian whose mean and covariance match those of the mixture.

### 3.3. RBPMF Propagation

The interacted RBPMF representation in Equation (38) is propagated using the conventional RBPMF operator [8,10]:

(38) $$D_{0,k}^{j}={\left\{\xi_{0,k}^{\left[g\right]},w_{0,k}^{j,[g]},{\hat{x}}_{0,k}^{j,l,[g]},P_{0,k}^{j,l,[g]}\right\}}_{g=1}^{G},$$

(39) $$D_{k+1|k}^{j}=T_{RBPMF}\left(D_{0,k}^{j};f_{k}^{n},F_{k}^{n},f_{k}^{l},F_{k}^{l},Q_{k}\right).$$

Because the navigation dynamics are common to all radar-return modes, the same propagation operator is applied to every destination mode. The resulting

$${\left\{\left(\mu_{k+1|k}^{j},\;D_{k+1|k}^{j}\right)\right\}}_{j=1}^{M}$$

forms the prior representation for the next measurement epoch. For a fixed state dimension and interpolation stencil, the additional interaction cost is approximately $O\left(M^{2}G\right)$.

## 4. Simulation Setup

Thirty Monte Carlo runs were performed to evaluate the proposed IMM-RBPMF. For the main comparisons, a bounded ground-return probability profile $\mu_{G}\left(k\right)\in [0,\;1]$, generated from a first-order Gauss–Markov process with $\tau_{c}=100$ s and σ=0.7, was fixed across both trajectories and all runs to isolate the effect of terrain informativeness. At each epoch, the return type was sampled for each run according to $\mu_{G}\left(k\right)$, and the radar-altimeter error was drawn from $N\left(0\;m,{\left(4.6\;m\right)}^{2}\right)$ for a ground return or $N\left(17\;m,{\left(9.1\;m\right)}^{2}\right)$ for a canopy return [14]. In these profile-driven simulations, the return sequence was not generated by the IMM transition matrix, which was used only as a fixed filter-side persistence prior.

Two 1200 s trajectories were considered. The DEM surfaces and flight paths are shown in Figure 3, and the corresponding terrain-elevation and local-roughness profiles are shown in Figure 4. Trajectory 1 traverses predominantly rough terrain, whereas Trajectory 2 includes a weakly informative flat-terrain segment from 200 to 800 s. Their initial geodetic positions, expressed as latitude, longitude, and altitude, were [36.2° N, 126.6583° E, 2000 m] and [36.6667° N, 127.0° E, 2000 m], respectively. For both trajectories, the prescribed roll, pitch, and heading were fixed at $\left[0^{\circ },0^{\circ },30^{\circ }\right]$. In a trajectory-fixed horizontal frame whose along-track axis was oriented at $30^{\circ }$ from north, the along-track velocity was 60 m/s, while the cross-track velocity was prescribed as $v_{⊥}\left(t\right)=60sin\left(2\pi \cdot 0.01t\right)\;m/s$. The resulting velocity vector was transformed into east–north–up coordinates. This 100 s-period kinematic maneuver provided repeated lateral excitation, with a peak cross-track acceleration of $3.77\;m/s^{2}$, or approximately 0.38 g. The translational motion and attitude were prescribed independently as an idealized kinematic benchmark, rather than generated by an aircraft flight-dynamics model. The resulting motion is therefore not intended to represent a coordinated fixed-wing turn. Separately, the radar-altimeter line of sight was assumed to remain aligned with the local vertical, excluding off-nadir ranging and attitude-dependent footprint variations. The evaluation is limited to these prescribed motion and sensing assumptions and does not establish performance under coupled aircraft and sensor dynamics. The local terrain roughness in Figure 4b was calculated as the standard deviation of bilinearly interpolated DEM elevations within ±75 m at 1.5 m spacing.

The proposed filter was compared with a ground-model RBPMF (RBPMF1), a canopy-model RBPMF (RBPMF2), and a fixed-mixture RBPMF employing a two-component Gaussian-mixture measurement model with weights [0.5, 0.5] (GM-RBPMF). The fixed weights [0.5, 0.5] assign equal weight to the two return hypotheses without using the prescribed ground-return probability profile. This baseline represents a nonadaptive mixture likelihood; the selected weights are not claimed to be optimal. The three methods are hereafter collectively referred to as the baseline filters; each maintains a single RBPMF posterior without IMM interaction. These RBPMF-based baselines were selected to assess the overall benefit of the IMM extension within a common state-estimation framework. The comparisons therefore contrast fixed single-return and fixed-mixture measurement models with recursive mode adaptation and interaction of mode-conditioned posteriors. All filters used the same navigation model and error-feedback configuration. Within each Monte Carlo run, identical initial-error and sensor-noise realizations and the same realized radar-return sequence were used across filters. To provide comparable total numbers of nonlinear grid points, the IMM-RBPMF used a 101 × 101 grid per mode, whereas each baseline filter used a 141 × 141 grid. The main simulation and filter parameters are summarized in Table 1.

The gyroscope and accelerometer biases listed in Table 1 are included in the simulated inertial measurement unit (IMU) errors but are not estimated as separate states. The nine-state formulation is a reduced-order approximation and is not equivalent to an augmented INS error model with explicit sensor-bias states. Performance was assessed using Monte Carlo root-mean-square errors (RMSEs) of the position, velocity, and attitude errors. For the flat-terrain segment of Trajectory 2, the temporal mean, 95th percentile, and maximum of the epoch-wise three-dimensional position RMSE were additionally evaluated. To quantify run-to-run variability, horizontal-position and altitude RMSEs were also calculated separately for each Monte Carlo run over the full trajectories, together with the 3D-position RMSE over the 200–800 s segment of Trajectory 2. The resulting run-wise RMSEs were summarized by their mean and sample standard deviation across the 30 runs. The number of runs in which the IMM-RBPMF achieved a lower RMSE than each baseline was also recorded using paired comparisons under identical input realizations.

Mode adaptation was assessed using the deviation between the Monte Carlo-averaged ground-mode probability and the prescribed profile $\mu_{G}\left(k\right)$, and computational cost was measured by the average processing time per update. Processing time was measured in MATLAB R2022b (The MathWorks, Inc., Natick, MA, USA) on a desktop computer equipped with an Intel Core i7-14700 processor (Intel Corporation, Santa Clara, CA, USA) and 32 GB RAM.

Additional Markov-switching validation was performed on Trajectory 1 using 30 Monte Carlo runs over the full 1200 s duration. The realized return mode at each epoch was sampled conditionally on the preceding mode using a data-generating transition matrix $\Pi_{true}$. Both $\Pi_{true}$ and the filter-side matrix $\Pi_{filter}$ were symmetric, with diagonal entries p and off-diagonal entries 1−p. Two matched cases, $\left(p_{true},p_{filter}\right)=\left(0.80,0.80\right)$ and $\left(0.95,0.95\right)$, and one mismatched case, $\left(0.95,0.80\right)$, were evaluated. For $p_{true}=0.95$, identical realized return sequences, initial errors, and sensor-noise realizations were used for both filter settings. The remaining simulation and filter settings in Table 1, including the 101×101 grid per mode, were unchanged. Mode-estimation performance was evaluated using the Brier score (BS) [25] and classification error rate (CER) against the realized binary return labels. BS evaluates the estimated mode probabilities using a strictly proper scoring rule, whereas CER evaluates the corresponding binary decisions [26].

## 5. Results and Discussion

Figure 5 compares the prescribed ground-return probability profile with the deviation of the Monte Carlo-averaged ground-mode estimate from that profile. Let ${\hat{\mu }}_{k,r}^{G}$ denote the estimated ground-mode probability at epoch k in the r-th Monte Carlo run. The profile-tracking deviation is defined as

(40) $$e_{\mu }\left(k\right)=|\frac{1}{N_{MC}}\sum_{r=1}^{N_{MC}}{\hat{\mu }}_{k,r}^{G}-\mu_{G}\left(k\right)|.$$

Because $\mu_{G}\left(k\right)$ is the probability used to generate the return type rather than the realized binary return label, $e_{\mu }\left(k\right)$ in Equation (40) should not be interpreted as a samplewise classification error. The time-averaged values of $e_{\mu }\left(k\right)$ were 0.096 and 0.094 for Trajectories 1 and 2, respectively, indicating comparable profile-tracking deviations for the two trajectories.

Figure 6 and Figure 7 show the horizontal-position and altitude RMSE histories, and Table 2 summarizes the overall RMSEs computed over the full trajectories. For Trajectory 1, the IMM-RBPMF achieved horizontal-position and altitude RMSEs of 5.6 and 2.2 m, compared with the best baseline values of 8.4 and 4.7 m, respectively. These results correspond to reductions of approximately 33% and 53%. For Trajectory 2, the horizontal-position RMSE decreased from the best baseline value of 19.6 m to 10.9 m, a reduction of approximately 44%, while the altitude RMSE was again reduced to 2.2 m. The velocity and attitude improvements were smaller because these states are corrected indirectly through INS/TRN coupling; nevertheless, the proposed filter produced lower or comparable RMSEs for all reported state components.

The largest performance difference occurs during the 200–800 s flat-terrain segment of Trajectory 2, where the small elevation variation and low local terrain roughness shown in Figure 4 weaken horizontal-position observability. At each epoch, the three-dimensional position RMSE was calculated over the Monte Carlo runs, and Table 3 reports the temporal mean, 95th percentile, and maximum of this RMSE sequence over 200–800 s. A comparison of Figure 6b and Figure 7b shows that the altitude RMSE remains broadly similar between the two trajectories, whereas Figure 7a exhibits pronounced horizontal-position error growth during the flat-terrain segment, particularly for the baseline filters. This contrast indicates that the large position excursions are primarily associated with horizontal-position error growth under weak terrain information, while the altitude RMSE remains comparatively limited. Although accurate altitude estimation remains important because the terrain-height measurement couples altitude and horizontal position, the GM-RBPMF results indicate that a small altitude error alone is insufficient to ensure horizontal-position robustness under weak or ambiguous terrain information. The reduced excursions of the IMM-RBPMF are therefore consistent with the joint effect of recursive mode adaptation and deterministic interaction of the complete mode-conditioned RBPMF representations.

The IMM-RBPMF reduces the mean, 95th-percentile, and maximum position RMSEs from the best baseline values of 23.3, 50.7, and 83.8 m to 12.4, 20.4, and 23.1 m, respectively. These values correspond to reductions of approximately 47%, 60%, and 72%. The results demonstrate that the proposed filtering framework maintains accurate altitude estimation while suppressing large horizontal-position excursions under weak terrain information and ambiguous radar-return conditions. This improvement should be interpreted as the integrated effect of the classification-error-minimizing mode update adopted from [14] and the proposed deterministic interaction of mode-conditioned RBPMF representations. The contributions of these two components are further examined through the ablation study presented later in this section.

Table 4 complements the aggregated performance measures in Table 2 and Table 3 by quantifying run-to-run variability in the principal position-error metrics.

The run-wise statistics in Table 4, together with paired comparisons, support the consistency of the position-accuracy advantage across the tested Monte Carlo realizations. The IMM-RBPMF yielded lower horizontal-position RMSE than each baseline in 29 of 30 runs for Trajectory 1 and in all 30 runs for Trajectory 2. Altitude RMSE was lower in every run of both trajectories, as was flat-segment 3D-position RMSE in every run of Trajectory 2. Thus, the improvement is not confined to a small number of favorable trials.

To assess the contributions of the truncated-likelihood mode update and grid-resolved posterior interaction, an ablation study was conducted on Trajectory 2, which includes the weakly informative flat-terrain segment. The proposed filter was compared with two variants: an IMM-RBPMF using the standard marginalized likelihood for mode-evidence evaluation, and a non-interacting multiple-model RBPMF (MM-RBPMF) retaining the truncated-likelihood mode update. In the latter, cross-mode posterior mixing was disabled, while mode-probability prediction and updating and the final mode-probability-weighted estimate were retained. All three configurations used the same grid size per mode and Monte Carlo input realizations, without retuning the remaining parameters. The ordinary marginalized likelihood used for navigation-state updating was unchanged.

Table 5 shows that replacing the truncated likelihood with the standard likelihood increased the horizontal-position and altitude RMSEs from 10.9 and 2.2 m to 18.1 and 5.8 m, respectively. Disabling posterior interaction while retaining the truncated-likelihood mode update increased these RMSEs to 19.6 and 7.0 m. Thus, with truncated-likelihood mode updating retained, enabling grid-resolved interaction reduced the horizontal-position and altitude RMSEs by approximately 44% and 69%, respectively. Conversely, with interaction retained, truncated-likelihood mode updating reduced these RMSEs by approximately 40% and 62% relative to standard mode-evidence evaluation. These comparisons show that the observed accuracy improvement is not attributable to the truncated-likelihood update alone; posterior interaction provides an additional benefit under the tested conditions. An additional 30-run comparison on Trajectory 2 used a 125 × 125 grid per mode for the non-interacting MM-RBPMF. Its average processing time was 0.797 s per update, compared with 0.766 s for the proposed filter using a 101 × 101 grid per mode. The horizontal-position and altitude RMSEs were 18.5 and 7.0 m, respectively, compared with 10.9 and 2.2 m for the proposed filter, indicating that the accuracy advantage was retained at comparable processing times under the tested conditions.

Table 6 summarizes the additional Markov-switching validation. Let $z_{k,r}=1$ denote a realized ground return and $z_{k,r}=0$ a canopy return at epoch k in run r. Using the filtered ground-mode probability ${\hat{\mu }}_{k,r}^{G}$, the binary decision is ${\hat{z}}_{k,r}=1\left\{{\hat{\mu }}_{k,r}^{G}\geq 0.5\right\}$. Over $N_{t}$ evaluated epochs per run, the scores are defined as

(41) $$BS=\frac{1}{N_{MC}N_{t}}\sum_{r=1}^{N_{MC}}\sum_{k=1}^{N_{t}}{\left({\hat{\mu }}_{k,r}^{G}-z_{k,r}\right)}^{2},$$

(42) $$CER=\frac{1}{N_{MC}N_{t}}\sum_{r=1}^{N_{MC}}\sum_{k=1}^{N_{t}}1\left\{{\hat{z}}_{k,r}\neq z_{k,r}\right\}.$$

Here, $1\left\{\cdot \right\}$ is the indicator function. These scores are calculated for each run and then averaged across runs; CER is reported as a percentage.

For the common $p_{true}=0.95$ data, the matched transition prior yielded lower BS, CER, and altitude RMSE than the nominal $p_{filter}=0.80$ prior, while horizontal-position RMSE was unchanged at the reported precision. The lower mode-estimation errors of Case B relative to Case A should not be interpreted as a benefit of mismatch, because the data-generating switching statistics also differed. Thus, the tested transition-prior mismatch had a limited impact on horizontal-position and altitude RMSEs in absolute terms.

To assess support-truncation loss separately from navigation RMSE, the total source-PMF mass at or below $\varepsilon_{w}/G$ was monitored before remapping and renormalization. For the nominal 101×101 grids, its mean was approximately $2.20\times 10^{-6}$ for Trajectory 1 and $2.33\times 10^{-6}$ for Trajectory 2 in both source modes. Across both trajectories and all Monte Carlo runs, the maximum values were $7.70\times 10^{-6}$ and $7.39\times 10^{-6}$ for the ground- and canopy-return modes, respectively. These values imply that at least 99.9992% of the represented source-PMF mass was retained during support truncation. They do not, however, bound interpolation-induced mean or covariance errors.

Sensitivity to the support-threshold parameter was additionally evaluated on Trajectory 1 using 30 Monte Carlo runs and a 101 × 101 grid per mode. Increasing $\epsilon_{w}$ from $10^{-4}$ to $10^{-3}$, with all other settings unchanged, changed the horizontal-position and altitude RMSEs from 5.6 and 2.2 m to 5.4 and 2.2 m, respectively. These results indicate limited sensitivity of the navigation RMSEs to this tenfold increase in the threshold under the tested conditions.

Figure 8 presents the trade-off between three-dimensional position RMSE and average processing time per update for Trajectory 1. The IMM-RBPMF used 51×51, 71×71, 101×101, and 121×121 grids per mode, whereas the baseline filters used 71×71, 101×101, 141×141, and 171×171 grids, respectively. The support threshold parameter was held fixed at $\varepsilon_{w}=10^{-4}$ for all IMM-RBPMF configurations in Figure 8. Runtime increased with grid size, and the IMM-RBPMF incurred additional cost for source-to-target remapping and Gaussian moment matching. Nevertheless, the IMM-RBPMF achieved lower three-dimensional position RMSE for all tested grid configurations. Although the absolute processing times depend on the MATLAB implementation and computing platform, the results provide an empirical characterization of the overall accuracy–cost trade-off across the tested grid configurations.

For the nominal two-mode configuration with a 101×101 grid per mode, the mean processing time was 0.766 s per update. The persistent storage of the two mode-conditioned filter objects was estimated as approximately 29.03 MiB by summing the sizes of their stored array properties. This estimate excludes temporary working arrays, DEM/input data, and result-storage arrays. The 20 Hz rate in Table 1 specifies a simulation update interval of 0.05 s, rather than an achieved execution rate. The current MATLAB implementation is intended for offline algorithmic evaluation and does not meet the 20 Hz real-time computational requirement on the tested platform.

## 6. Conclusions

This paper extended the interacting multiple-model (IMM) framework to Rao–Blackwellized point-mass filtering for TRN under radar return ambiguity, with ground and canopy returns represented as alternative measurement modes. The extension retains the truncated-likelihood mode update developed in [14] and introduces grid-resolved posterior mixing, a deterministic interaction procedure for complete mode-conditioned RBPMF representations. During this interaction, the nonlinear-state PMF masses are remapped and mixed on a common target grid, while the associated grid-conditioned Gaussian components are combined using local responsibilities and moment matching. The resulting IMM-RBPMF incorporates recursive measurement-mode adaptation while retaining the deterministic grid-based representation of nonlinear-state uncertainty.

Monte Carlo simulations showed that the proposed filter achieved lower horizontal-position and altitude RMSEs than all evaluated single-model and fixed-mixture RBPMF baselines. Relative to the best-performing baseline for each metric and trajectory, it reduced horizontal-position RMSE by 33% and 44% for Trajectories 1 and 2, respectively, and altitude RMSE by 53% in both cases. Over the flat-terrain segment, the temporal 95th percentile of the epoch-wise three-dimensional position RMSE was reduced by 60%, demonstrating effective suppression of large position-error excursions under weakly informative terrain and radar return ambiguity. Although grid-resolved interaction introduced additional computation, the proposed filter achieved lower three-dimensional position RMSE across all tested grid configurations.

## Figures and Tables

**Figure 1:**
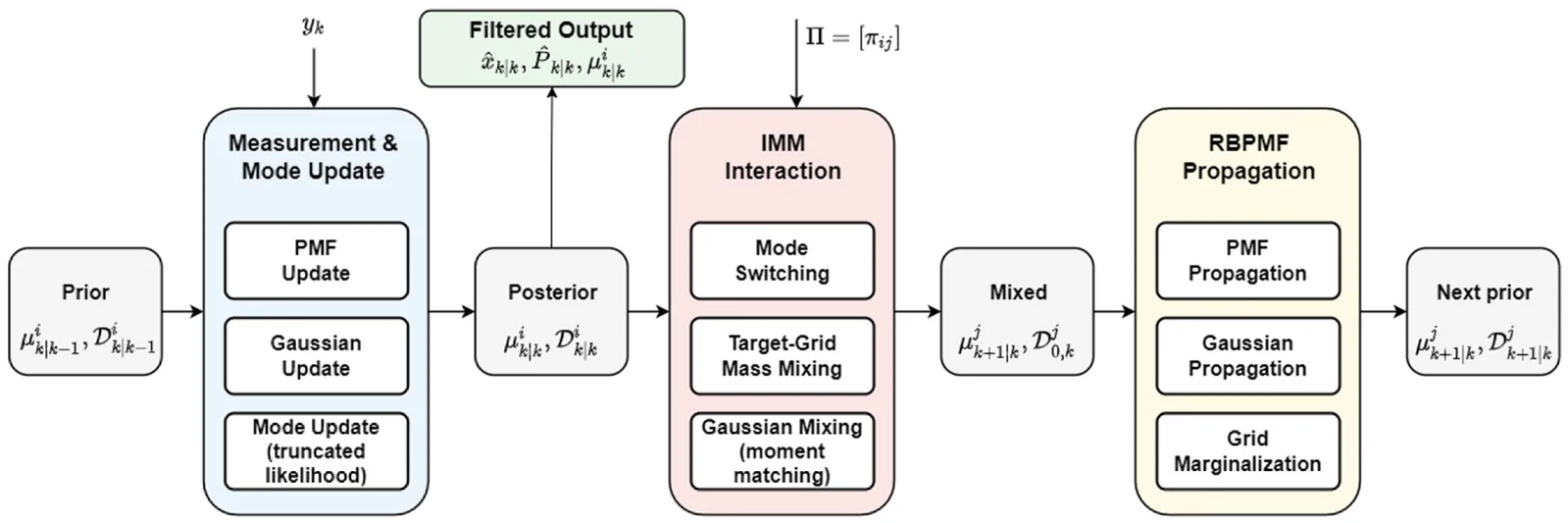
Overall flow of the proposed IMM-RBPMF recursion. Colors are used to visually distinguish the processing stages, and arrows indicate the direction of information flow. Abbreviations: IMM, interacting multiple model; PMF, point-mass filter; RBPMF, Rao–Blackwellized point-mass filter.

**Figure 2:**
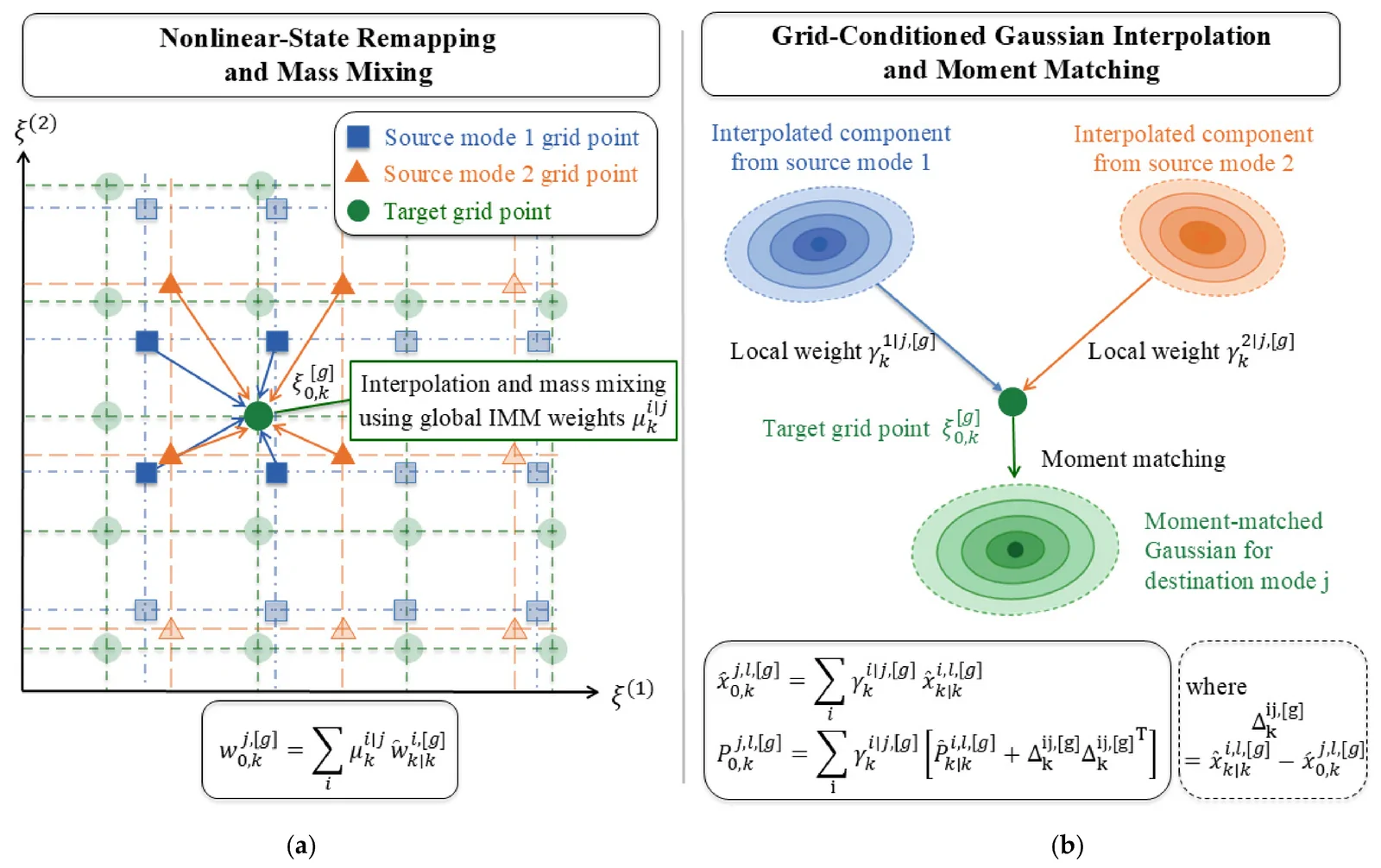
Deterministic implementation of RBPMF interaction for each destination mode via grid-resolved posterior mixing. (**a**) Source-mode PMF masses are converted to density-equivalent values, interpolated onto the target grid, renormalized as target-grid masses, and mixed using the global IMM interaction probabilities. The dashed colored lines indicate the source- and target-grid structures, and the arrows indicate interpolation and mixing contributions to the target grid point. (**b**) The grid-conditioned Gaussian components transferred to the same target-grid point are combined using local responsibilities and reduced to a single moment-matched Gaussian component for each destination mode. The shaded ellipses schematically represent the Gaussian components and their dispersion.

**Figure 3:**
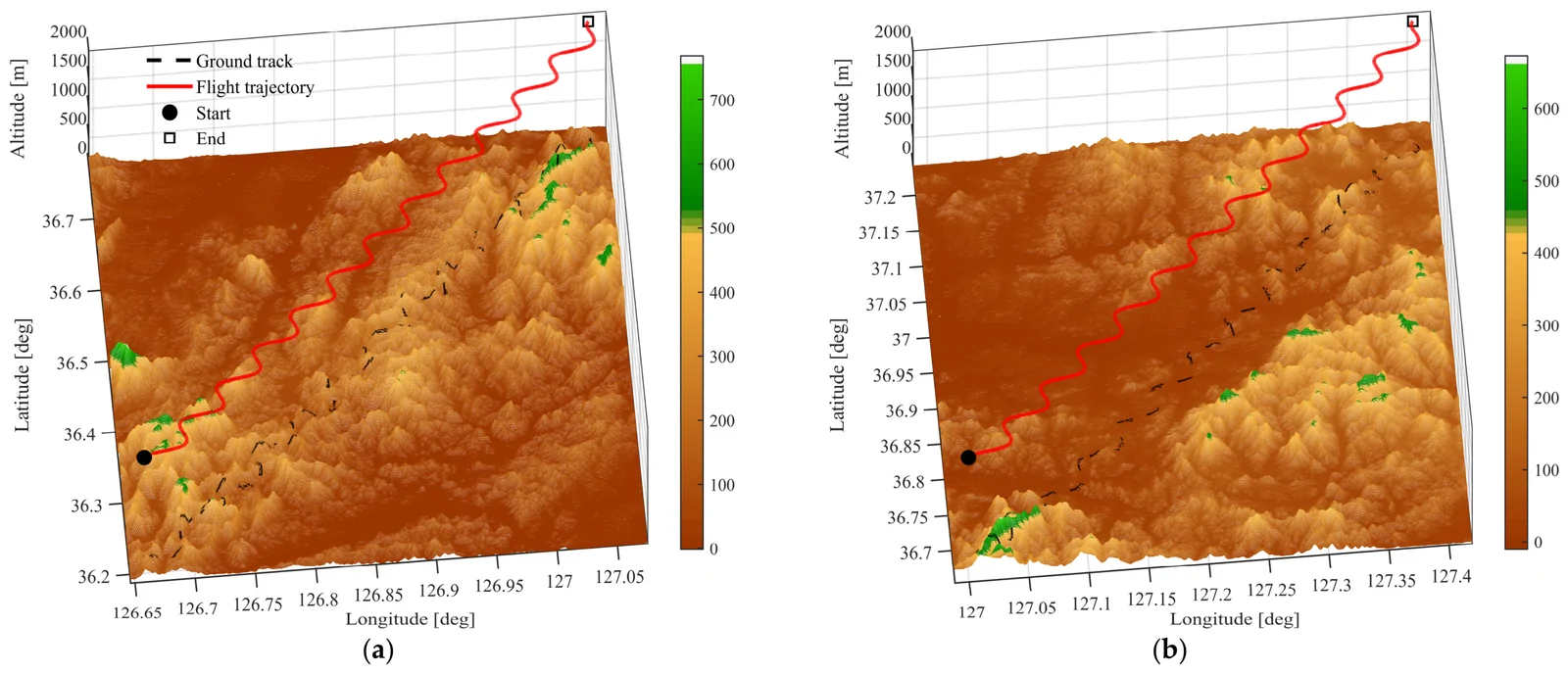
Digital elevation model (DEM) surfaces and flight trajectories used in the simulations. (**a**) Trajectory 1 over predominantly rough terrain. (**b**) Trajectory 2 over rough–flat–rough terrain. In both panels, the red solid line denotes the flight trajectory at a constant altitude of 2000 m, the dashed line denotes the ground-track projection on the terrain surface, and the circle and square markers indicate the start and end points, respectively.

**Figure 4:**
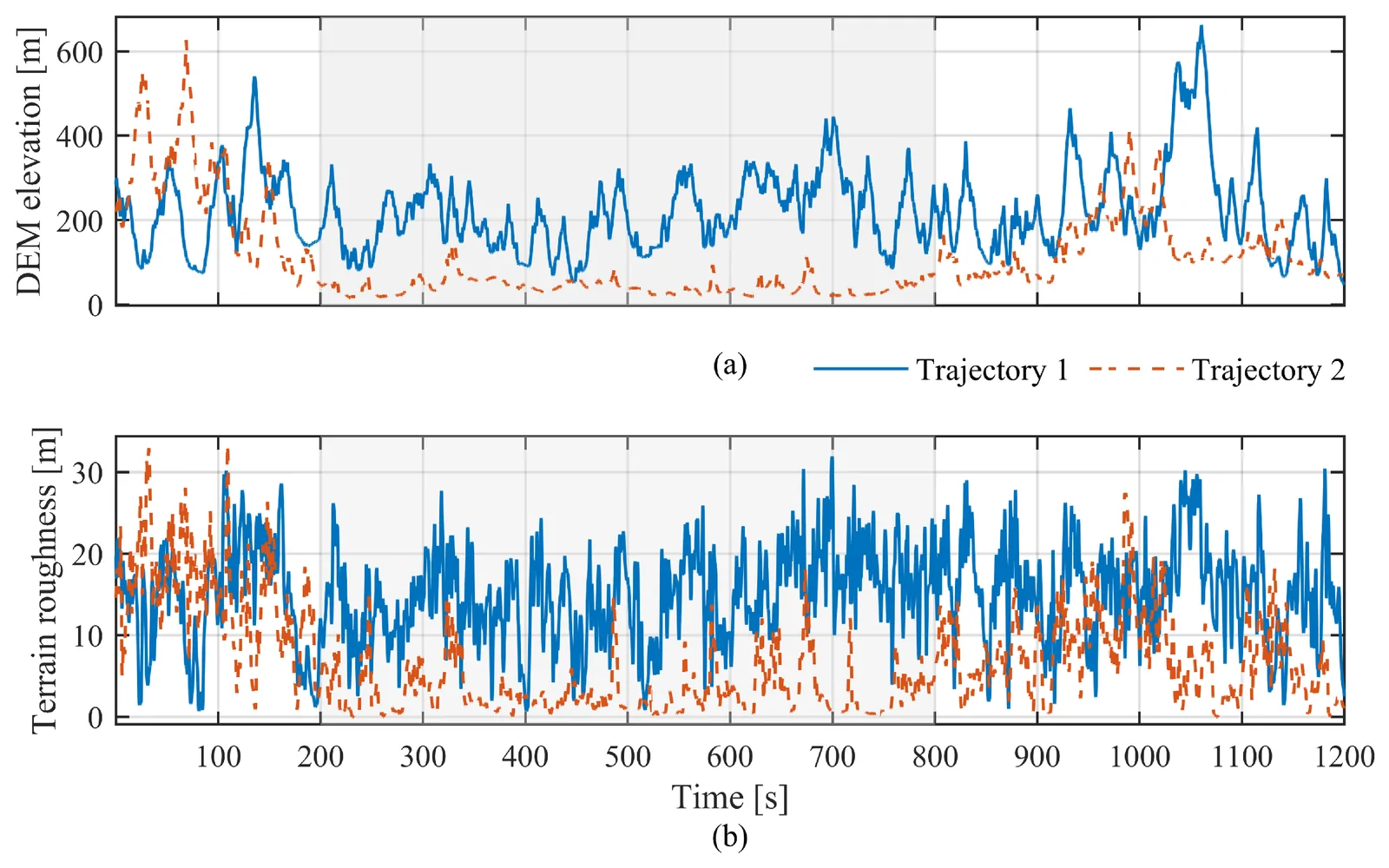
Terrain characteristics along the simulation trajectories. (**a**) DEM elevation along the trajectories. (**b**) Local terrain roughness along the trajectories. The shaded interval denotes the 200–800 s flat-terrain segment of Trajectory 2.

**Figure 5:**
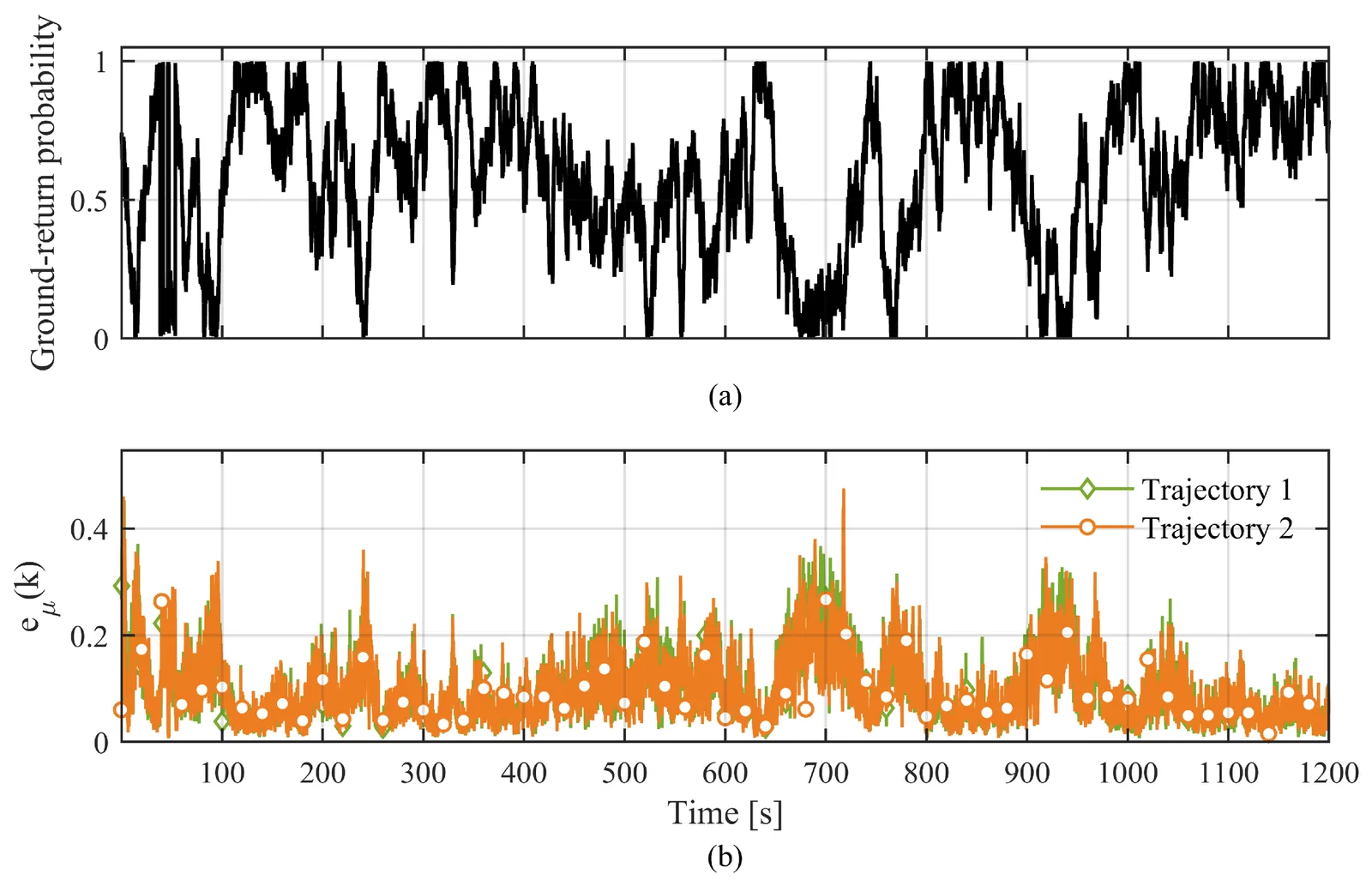
Prescribed ground-return probability profile and mode-probability tracking performance. (**a**) Prescribed ground-return probability profile $\mu_{G}\left(k\right)$, shown by the black line. (**b**) Profile-tracking deviation $e_{\mu }\left(k\right)$ for Trajectories 1 and 2.

**Figure 6:**
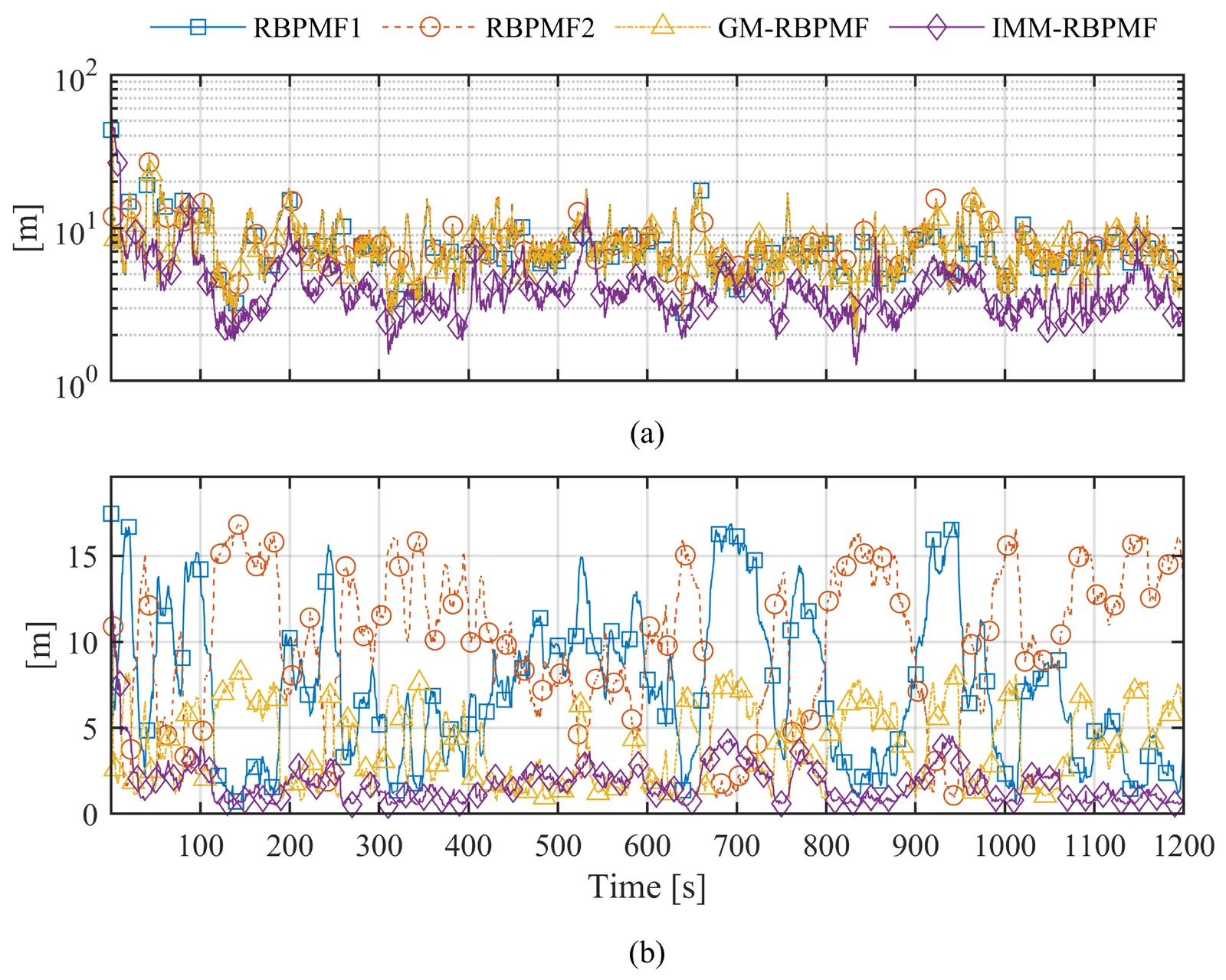
Root-mean-square error (RMSE) histories of (**a**) horizontal position and (**b**) altitude for Trajectory 1.

**Figure 7:**
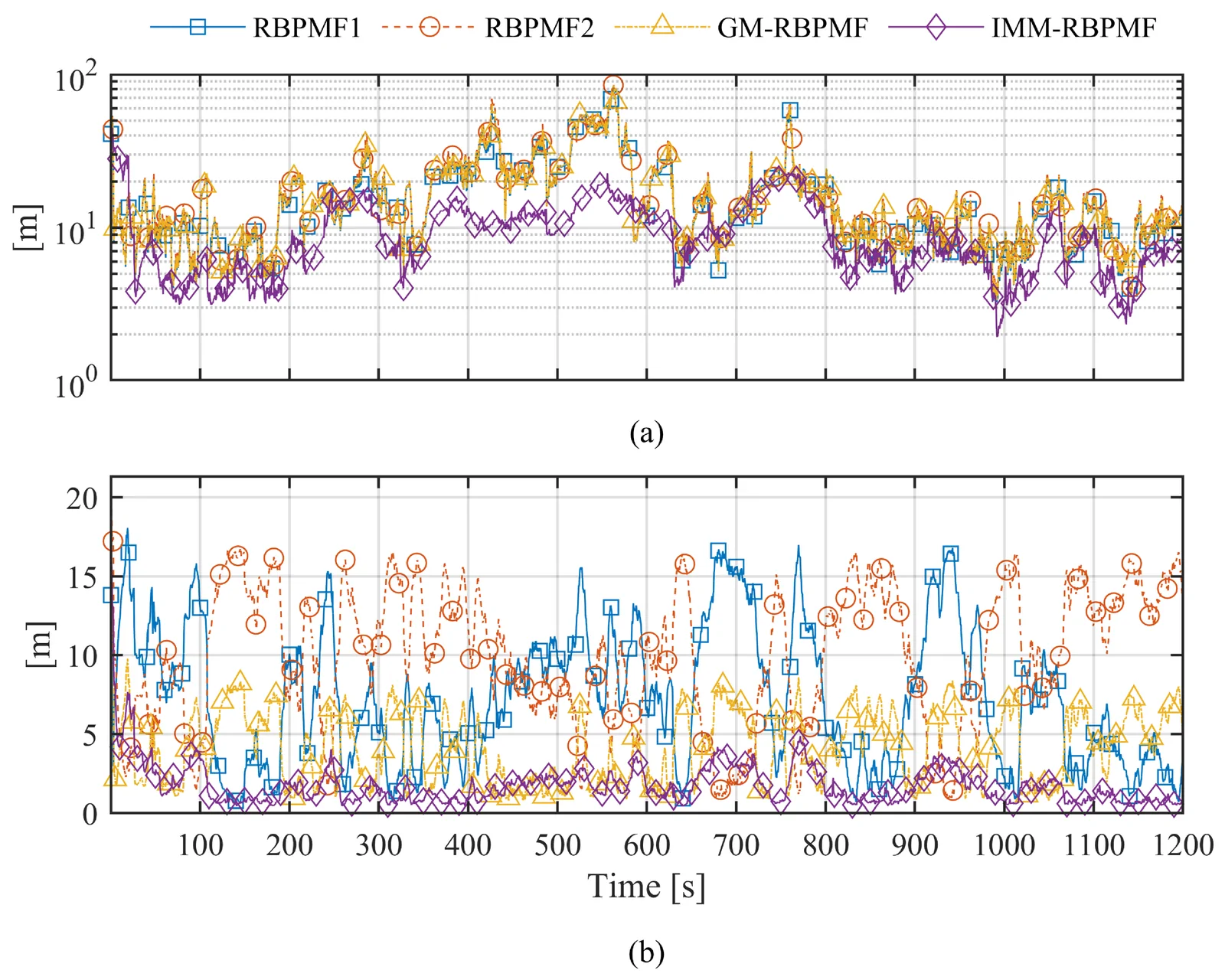
RMSE histories of (**a**) horizontal position and (**b**) altitude for Trajectory 2.

**Figure 8:**
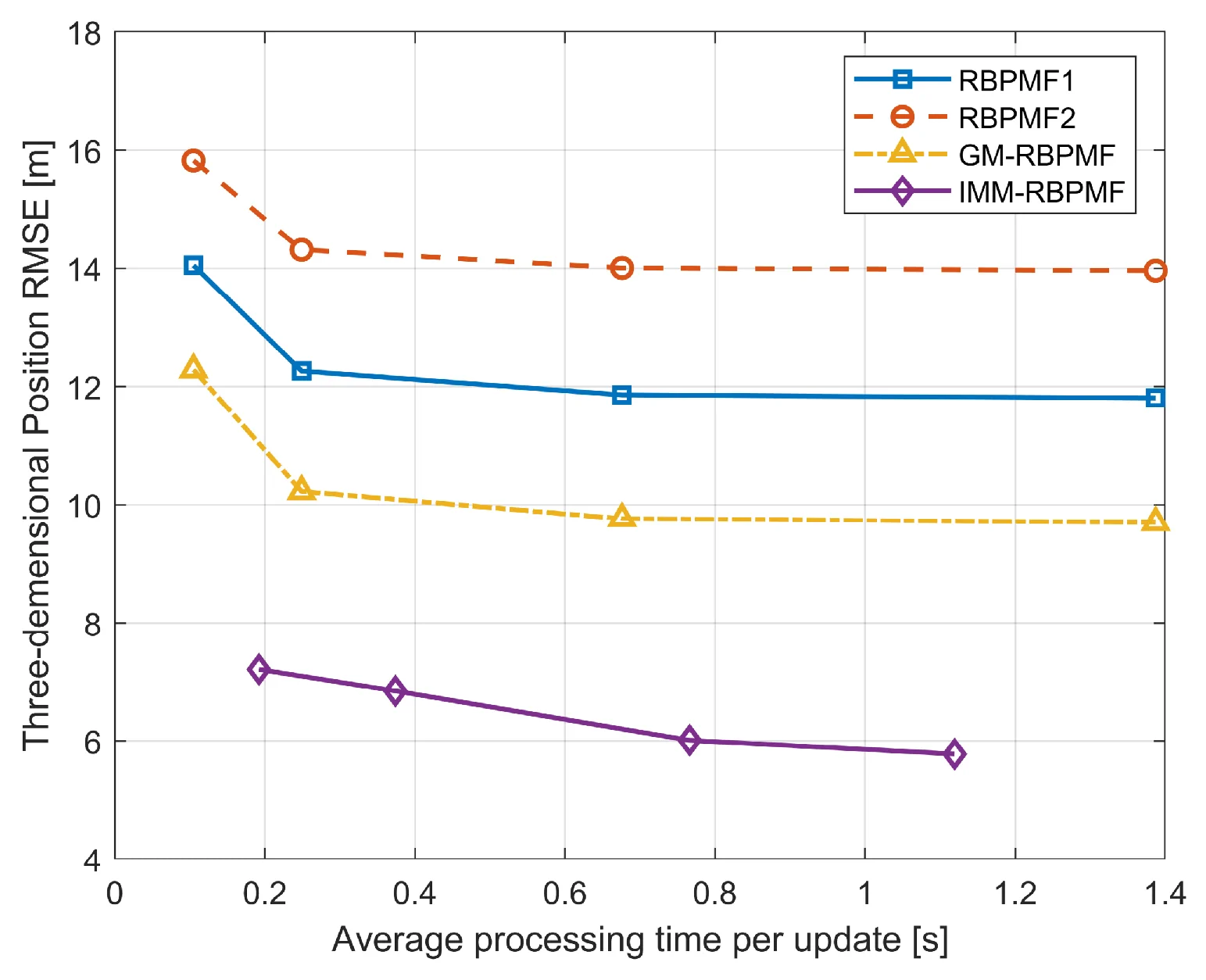
Three-dimensional position RMSE versus average processing time per update for Trajectory 1.

**Table 1:** Main simulation parameters.

Parameter	Value
Simulated filter update rate/DEM resolution	20 Hz/10 m
INS grade	1 nmi/h with barometric-altimeter damping
Initial INS errors $\left(1\sigma \right)$	Position [20, 20, 20] m, Velocity [0.5, 0.5, 0.5] m/s, Attitude [0.3, 0.3, 1.5] mrad
Gyro/Accelerometer bias $\left(1\sigma \right)$	0.01 deg/h/100 μg
Barometric-altimeter bias $\left(1\sigma \right)$	20 m
Barometric-altimeter Markov noise	$\tau_{c}=20\;s,\;\sigma =1\;m$
Ground-return probability profile	$\mu_{G}(k)$: Gauss-Markov-driven probability profile, $\tau_{c}=100\;s,$ σ=0.7
Radar error models	Ground: $N(0\;m,{\left(4.6\;m\right)}^{2}),$ canopy: $N(17\;m,{\left(9.1\;m\right)}^{2})$
Nominal IMM parameters	$\mu_{0}=[0.9;0.1],\;\Pi =[0.8\;0.2;0.2\;0.8]$
Grid size	IMM-RBPMF: 101×101 per mode, Baseline RBPMFs: 141×141

**Table 2:** Overall navigation-error RMSEs computed over all epochs and Monte Carlo runs for Trajectories 1 and 2.

Trajectory	Metric (RMSE)	RBPMF1	RBPMF2	GM-RBPMF	IMM-RBPMF
Trajectory 1	Hor. Pos. [m]	8.4	8.8	8.6	5.6
	Altitude [m]	8.4	10.9	4.7	2.2
	Hor. Vel. [m/s]	0.23	0.23	0.23	0.18
	Ver. Vel. [m/s]	0.11	0.10	0.11	0.09
	Hor. Att. [mrad]	0.22	0.22	0.22	0.21
	Ver. Att. [mrad]	0.95	0.95	0.95	0.82
Trajectory 2	Hor. Pos. [m]	19.6	20.7	20.1	10.9
	Altitude [m]	8.5	10.9	4.7	2.2
	Hor. Vel. [m/s]	0.31	0.31	0.31	0.23
	Ver. Vel. [m/s]	0.11	0.11	0.11	0.10
	Hor. Att. [mrad]	0.23	0.23	0.23	0.22
	Ver. Att. [mrad]	1.16	1.16	1.16	1.12

Note: Hor., horizontal; Ver., vertical; Pos., position; Vel., velocity; Att., attitude.

**Table 3:** Three-dimensional position RMSE statistics over the flat-terrain segment of Trajectory 2 from 200 to 800 s.

Filter	Mean Position RMSE [m]	95th-Percentile Position RMSE [m]	Maximum Position RMSE [m]
RBPMF1	24.3	50.7	83.8
RBPMF2	25.8	52.1	86.2
GM-RBPMF	23.3	51.1	84.7
IMM-RBPMF	12.4	20.4	23.1

**Table 4:** Run-wise position RMSE statistics across Monte Carlo runs.

Trajectory	Filter	Horizontal-Position RMSE [m]	Altitude RMSE [m]	Flat-Segment 3D RMSE [m]
Trajectory 1	RBPMF1	8.37 ± 0.37	8.39 ± 0.23	–
	RBPMF2	8.79 ± 0.44	10.89 ± 0.24	–
	GM-RBPMF	8.55 ± 0.40	4.70 ± 0.14	–
	IMM-RBPMF	5.43 ± 1.42	2.15 ± 0.33	–
Trajectory 2	RBPMF1	19.46 ± 2.11	8.45 ± 0.18	26.99 ± 3.03
	RBPMF2	20.54 ± 2.28	10.86 ± 0.13	28.59 ± 3.23
	GM-RBPMF	19.98 ± 2.19	4.71 ± 0.11	26.42 ± 3.28
	IMM-RBPMF	10.75 ± 1.59	2.16 ± 0.27	13.01 ± 1.81

Note: Values are the mean ± sample standard deviation of RMSEs calculated separately for each of the 30 Monte Carlo runs. Horizontal-position and altitude RMSEs are evaluated over the full trajectories; flat-segment 3D RMSEs are evaluated over 200–800 s of Trajectory 2. A dash indicates that the metric is not applicable.

**Table 5:** Ablation study on Trajectory 2: overall horizontal-position and altitude RMSEs.

Configuration	Mode-Evidence Likelihood	Posterior Interaction	Horizontal RMSE [m]	Altitude RMSE [m]
IMM-RBPMF (proposed)	Truncated	Enabled	10.9	2.2
IMM-RBPMF with standard likelihood	Standard	Enabled	18.1	5.8
Non-interacting MM-RBPMF	Truncated	Disabled	19.6	7.0

Note: RMSEs are computed over all epochs and Monte Carlo runs, as in Table 2. “Truncated” refers to the mode-evidence likelihood, not PMF support truncation.

**Table 6:** IMM-RBPMF performance under Markov-generated radar returns and transition-prior mismatch on Trajectory 1.

Case	$p_{true}$	$p_{filter}$	BS	CER [%]	Horizontal RMSE [m]	Altitude RMSE [m]
A: Matched	0.80	0.80	0.088	10.35	5.3	1.9
B: Mismatched	0.95	0.80	0.069	8.23	5.3	1.8
C: Matched	0.95	0.95	0.055	6.41	5.3	1.6

Note: $p_{true}$ and $p_{filter}$ are the self-transition probabilities of the symmetric data-generating and filter-side transition matrices, respectively. Cases B and C use identical input realizations within each Monte Carlo run. BS and CER are averaged over the 30 runs; position RMSEs are computed over all epochs and runs, as in Table 2.

## Data Availability

The data supporting the findings of this study are not publicly available due to institutional restrictions on external data sharing.

## Abbreviations

The following abbreviations are used in this manuscript:

DEM	Digital elevation model
GM-RBPMF	Gaussian-mixture Rao–Blackwellized point-mass filter
GNSS	Global navigation satellite system
IMM	Interacting multiple model
IMM-PF	Interacting multiple-model particle filter
IMM-PMF	Interacting multiple-model point-mass filter
IMM-RBPMF	Interacting multiple-model Rao–Blackwellized point-mass filter
INS	Inertial navigation system
MMAE	Multiple-model adaptive estimation
PF	Particle filter
PMF	Point-mass filter
RBPMF	Rao–Blackwellized point-mass filter
RMSE	Root-mean-square error
TRN	Terrain-referenced navigation

## Appendix A

The state partition follows Section 2.1. The horizontal-position errors are measured in meters, and the attitude errors are small-angle ENU components in radians:

$$x_{k}^{n}={\left[\delta p_{E,k},\delta p_{N,k}\right]}^{T},\;\;x_{k}^{l}={\left[\delta h_{k},\delta v_{E,k},\delta v_{N,k},\delta v_{U,k},\phi_{E,k},\phi_{N,k},\phi_{U,k}\right]}^{T}.$$

Let L, λ and h denote nominal latitude, longitude and altitude, respectively. The horizontal-position errors using the instantaneous nominal curvature scales are defined as

(A1) $$\delta p_{E}=r_{t}cosL\;\delta \lambda ,\;\;\delta p_{N}=r_{m}\;\delta L.$$

For compactness, define

(A2) $$r_{t}=R_{t}\left(L\right)+h,\;r_{m}=R_{m}\left(L\right)+h,\;\;\;\;\;\;\kappa_{t}=\frac{R_{t}^{\prime }\left(L\right)}{r_{t}},\;\kappa_{m}=\frac{R_{m}^{\prime }\left(L\right)}{r_{m}},\;\chi =tanL-\kappa_{t}.$$

Here, $R_{t}$ and $R_{m}$ are the prime-vertical and meridian curvature radii. A prime denotes a derivative with respect to *L*. With Ω denoting the Earth rotation rate, the Earth-rotation and transport-rate components are

(A3) $$\Omega_{N}=\Omega cosL,\;\Omega_{U}=\Omega sinL,\;\rho_{E}=-\frac{v_{N}}{r_{m}},\;\rho_{N}=\frac{v_{E}}{r_{t}},\;\rho_{U}=\rho_{N}tanL.$$

Perturbations of gravity are omitted, and sensor biases are not included as estimated states. All coefficients are evaluated at time k, with their time subscripts omitted, and the transition blocks are obtained by first-order discretization with update interval Δt.

The nonlinear-state transition blocks are

(A4) $$A_{k}^{nn}=I_{2}+\Delta t\left[\begin{array}{cc} \frac{v_{U}}{r_{t}}-\frac{v_{N}\chi }{r_{m}} & \frac{v_{E}\chi }{r_{m}}\\ 0 & \frac{v_{U}}{r_{m}} \end{array}\right],$$

(A5) $$A_{k}^{nl}=\Delta t\left[\begin{array}{ccccccc} -\rho_{N} & 1 & 0 & 0 & 0 & 0 & 0\\ \rho_{E} & 0 & 1 & 0 & 0 & 0 & 0 \end{array}\right].$$

The diagonal terms in (A4) account for the variation in the angle-to-meter scales in (A1). The coupling from horizontal-position errors to the conditionally linear states is

(A6) $$A_{k}^{ln}=\frac{\Delta t}{r_{m}}\left[\begin{array}{cc} 0 & 0\\ 0 & \left(\rho_{N}{sec}^{2}L+2\Omega_{N}-\rho_{U}\kappa_{t}\right)v_{N}+\left(\rho_{N}\kappa_{t}+2\Omega_{U}\right)v_{U}\\ 0 & -\left(\rho_{N}{sec}^{2}L+2\Omega_{N}\right)v_{E}-\rho_{E}\kappa_{m}v_{U}+\rho_{N}\rho_{U}R_{t}^{\prime }\\ 0 & -\rho_{N}^{2}R_{t}^{\prime }-\rho_{E}^{2}R_{m}^{\prime }-2\Omega_{U}v_{E}\\ 0 & -\rho_{E}\kappa_{m}\\ 0 & -\Omega_{U}-\rho_{N}\kappa_{t}\\ 0 & \Omega_{N}+\rho_{N}{sec}^{2}L-\rho_{U}\kappa_{t} \end{array}\right].$$

For the state ordering $\left[\delta h,\delta v_{E},\delta v_{N},\delta v_{U},\phi_{E},\phi_{N},\phi_{U}\right]ᵀ$, the remaining block is

(A7) $$A_{k}^{ll}=I_{7}+\Delta t\left[\begin{array}{ccccccc} 0 & 0 & 0 & 1 & 0 & 0 & 0\\ \frac{-\rho_{U}v_{N}+\rho_{N}v_{U}}{r_{t}} & \frac{v_{N}tanL-v_{U}}{r_{t}} & 2\Omega_{U}+\rho_{U} & -2\Omega_{N}-\rho_{N} & 0 & -f_{U} & f_{N}\\ -\frac{\rho_{E}v_{U}}{r_{m}}+\rho_{N}\rho_{U} & -2\left(\Omega_{U}+\rho_{U}\right) & -\frac{v_{U}}{r_{m}} & \rho_{E} & f_{U} & 0 & -f_{E}\\ -\rho_{N}^{2}-\rho_{E}^{2} & 2\left(\Omega_{N}+\rho_{N}\right) & -2\rho_{E} & 0 & -f_{N} & f_{E} & 0\\ -\frac{\rho_{E}}{r_{m}} & 0 & -\frac{1}{r_{m}} & 0 & 0 & \Omega_{U}+\rho_{U} & -\Omega_{N}-\rho_{N}\\ -\frac{\rho_{N}}{r_{t}} & \frac{1}{r_{t}} & 0 & 0 & -\Omega_{U}-\rho_{U} & 0 & \rho_{E}\\ -\frac{\rho_{U}}{r_{t}} & \frac{tanL}{r_{t}} & 0 & 0 & \Omega_{N}+\rho_{N} & -\rho_{E} & 0 \end{array}\right].$$

The quantities $f_{E}$, $f_{N}$ and $f_{U}$ are the nominal specific-force components in ENU coordinates. These blocks specify the nine-state transition matrix $A_{k}$.

## Appendix B

The nonlinear-state-dependent local responsibility used in Equation (27) is derived as follows. Let $Y\equiv Y_{0:k}$, and define

(A8) $$\mu_{k}^{i}≜P\left(\theta_{k}=\theta^{i}|Y\right),$$

(A9) $$p_{i}^{n}\left(x_{k}^{n}\right)≜p\left(x_{k}^{n}|\theta_{k}=\theta^{i},Y\right),$$

(A10) $$p_{i}^{l}\left(x_{k}^{l}|x_{k}^{n}\right)≜p\left(x_{k}^{l}|x_{k}^{n},\theta_{k}=\theta^{i},Y\right).$$

Then, the mode-conditioned posterior is written as

(A11) $$p\left(x_{k}^{n},x_{k}^{l}|\theta_{k}=\theta^{i},Y\right)=p_{i}^{n}\left(x_{k}^{n}\right)p_{i}^{l}\left(x_{k}^{l}|x_{k}^{n}\right).$$

Assume that the Markov mode transition is independent of the continuous state:

(A12) $$P\left(\theta_{k+1}=\theta^{j}|\theta_{k}=\theta^{i},x_{k}^{n},x_{k}^{l},Y\right)=\pi_{ij}.$$

For destination mode $\theta_{k+1}=\theta^{j}$, the conditional linear-state density is

(A13) $$p\left(x_{k}^{l}|x_{k}^{n},\theta_{k+1}=\theta^{j},Y\right)=\sum_{i=1}^{M}p_{i}^{l}\left(x_{k}^{l}|x_{k}^{n}\right)P\left(\theta_{k}=\theta^{i}|x_{k}^{n},\theta_{k+1}=\theta^{j},Y\right)\;=\sum_{i=1}^{M}\gamma_{k}^{i|j}\left(x_{k}^{n}\right)p_{i}^{l}\left(x_{k}^{l}|x_{k}^{n}\right),$$

where

(A14) $$\gamma_{k}^{i|j}\left(x_{k}^{n}\right)≜P\left(\theta_{k}=\theta^{i}|x_{k}^{n},\theta_{k+1}=\theta^{j},Y\right).$$

Using Bayes’ rule,

(A15) $$\gamma_{k}^{i|j}\left(x_{k}^{n}\right)=\frac{P\left(\theta_{k+1}=\theta^{j}|\theta_{k}=\theta^{i},x_{k}^{n},Y\right)P\left(\theta_{k}=\theta^{i}|x_{k}^{n},Y\right)}{P\left(\theta_{k+1}=\theta^{j}|x_{k}^{n},Y\right)}\;=\frac{\pi_{ij}P\left(\theta_{k}=\theta^{i}|x_{k}^{n},Y\right)}{\sum_{r=1}^{M}\pi_{rj}P\left(\theta_{k}=\theta^{r}|x_{k}^{n},Y\right)}.$$

Since

(A16) $$P\left(\theta_{k}=\theta^{i}|x_{k}^{n},Y\right)=\frac{p_{i}^{n}\left(x_{k}^{n}\right)\mu_{k}^{i}}{p\left(x_{k}^{n}|Y\right)},$$

the common denominator $p\left(x_{k}^{n}|Y\right)$ cancels out. Therefore,

(A17) $$\gamma_{k}^{i|j}\left(x_{k}^{n}\right)=\frac{\pi_{ij}\mu_{k}^{i}p_{i}^{n}\left(x_{k}^{n}\right)}{\sum_{r=1}^{M}\pi_{rj}\mu_{k}^{r}p_{r}^{n}\left(x_{k}^{n}\right)}.$$

Equivalently, using the standard IMM interaction probability

(A18) $$\mu_{k}^{i|j}=\frac{\pi_{ij}\mu_{k}^{i}}{\sum_{r=1}^{M}\pi_{rj}\mu_{k}^{r}},$$

the responsibility can be written as

(A19) $$\gamma_{k}^{i|j}\left(x_{k}^{n}\right)=\frac{\mu_{k}^{i|j}p_{i}^{n}\left(x_{k}^{n}\right)}{\sum_{r=1}^{M}\mu_{k}^{r|j}p_{r}^{n}\left(x_{k}^{n}\right)}.$$

Thus, the dependence of $\gamma_{k}^{i|j}\left(x_{k}^{n}\right)$ comes from the mode-conditioned nonlinear-state density $p_{i}^{n}\left(x_{k}^{n}\right)$, not from a state-dependent Markov transition.

In the deterministic PMF implementation, let ${\tilde{w}}_{k|k}^{i,\left[g\right]}$ denote the normalized mass of source mode i after density-based remapping to target-grid point $\xi_{0,k}^{\left[g\right]}$. Because all source modes are represented on the same target grid, the common target-cell area cancels in the responsibility ratio. The discrete responsibility used in Equation (34) is then

(A20) $$\gamma_{k}^{i|j,\left[g\right]}=\frac{\mu_{k}^{i|j}{\tilde{w}}_{k|k}^{i,\left[g\right]}}{\sum_{r=1}^{M}\mu_{k}^{r|j}{\tilde{w}}_{k|k}^{r,\left[g\right]}},$$

Equivalently,

(A21) $$\gamma_{k}^{i|j,\left[g\right]}=\frac{\pi_{ij}\mu_{k}^{i}{\tilde{w}}_{k|k}^{i,\left[g\right]}}{\sum_{r=1}^{M}\pi_{rj}\mu_{k}^{r}{\tilde{w}}_{k|k}^{r,\left[g\right]}}.$$

This responsibility is used for the grid-wise moment matching of the conditional Gaussian components in Equations (35) and (36).
